# Challenges to assessing professional identity in medical students: a tale of two measures

**DOI:** 10.1080/10872981.2019.1649571

**Published:** 2019-08-07

**Authors:** Era Buck, Courtney West, Lori Graham, Ann W Frye, Cayla R Teal

**Affiliations:** a Family Medicine, Educational Development, UTMB, Galveston, TX, USA; b Academic Affairs, Sam Houston State University Proposed College of Osteopathic Medicine, Huntsville, TX, USA; c Internal Medicine, Texas A&M University Health Science Center, College of Medicine, Bryan, TX, USA; d Internal Medicine, Educational Development, The University of Texas Medical Branch, Galveston, TX, USA; e Academic Affairs, Texas A&M University Health Science Center, College of Medicine, Bryan, TX, USA

**Keywords:** Professional identity, assessment, validity, undergraduate medical education, identity

## Abstract

**Background**: Professional identity formation (PIF), a foundational process in becoming a physician, includes establishment of values, moral principles, and self-awareness. The purpose of this report is to examine challenges in establishing the validity of measures of identity fusion as one facet of PIF.

**Method**: Utilizing the modern approach of validity as a unitary concept, the authors generated six hypotheses to examine the evidence for the construct validity of the scores of Physician Professional Identity (PPI) and Identity Integration (IdIn), considering relationships of these measures with each other, year of training and data from a larger survey.

**Results**: Responses from 3473 students at 8 medical schools revealed a weak association between the measures with distributions varying by cohort. PPI had a stronger relationship to cohort and IdIn was moderately associated with students’ attitudes relevant to social media use. Responses were independent of response format and evidence supported the interpretation of scores for IdIn as indications of integration of identity.

**Discussion **: Sufficient evidence was found to suggest that these measures assess aspects of PIF. Use of these measures as part of a multidimensional, longitudinal approach to refining understanding of the construct of PIF and developing effective assessment strategies.

## Introduction

Professional identity formation (PIF) in medicine is a foundational process experienced during the transformation from college student to physician [1,2]. This integrative, developmental process involves the establishment of core values, moral principles, and self-awareness. Recent work by the Pediatric Milestone Project [3] considered identity formation to be an essential element of professionalism. Cohen [4] emphasized the importance of fostering humanism (‘a way of being’) rather than focusing on professionalism (‘a way of acting’) as observable behaviors. Rabow and colleagues [5], as well as Cooke and colleagues [1], proposed that medical education should move beyond instruction in ethics and elements of professionalism to a more holistic focus on the complexities of professional formation in students and physicians.

Despite the repeated emphasis on PIF in the medical education literature [6, 7; 8], few assessment methodologies have been identified and validated. Stern [9] outlined strategies that have been used to assess professionalism components. Many of these measurements depend on relatively brief observable behaviors and have limited utility for assessing the formation process. Other strategies used to investigate learners’ identity development include reflection [10], portfolios [11], and assessment of moral reasoning [12]. While these strategies have been evaluated as individual measures of professional behavior or of identity formation components, they provide, at best, an incomplete approach to assessing PIF. The complexity of the formation process and its inherently phenomenological nature require the adoption of multifaceted assessment strategies that include self-report instruments [13–15].

Better assessment tools are needed if medical educators are to be able to describe how identity formation takes place among medical students. The study of PIF is nascent, presenting both need and challenge for creating assessments. To describe PIF and to assess the impact of experiences on PIF, we need measurement tools. The creation of those tools would benefit from a clear conceptual framework that is currently absent for PIF. The co-evolution of the construct of PIF and the tools to assess that process will likely require an iterative cycle that includes assessment, consideration of data, analysis of process, refinement of assessment tools and then additional assessment, all in the context of the evolving knowledge base for PIF. Previously reported relevant assessments focused on the impact of curricular experiences [16] or identity formation in a particular clinical or cultural context [17,18].

The extent to which an individual considers him/herself part of a larger group, e.g., the extent to which medical students consider themselves members of physicians as a group [19], is one aspect of the formative process. Some work in this area refers to this aspect of the formation process as *identity fusion* [20]. Individuals experiencing identity fusion maintain both a strong personal identity and a strong identity with the group. PIF is a process relevant to both personal development and alignment with the profession. Personal and professional identities are symbiotic, reinforcing and strengthening each other. Identity fusion has been predictive of individuals’ willingness to make personal sacrifices for the benefit of the group such as is required of physicians whose sense of duty as a physician leads them to postpone personal gratification sometimes putting aside their own needs [21]. Thus, identity fusion is a useful construct in understanding the complex process of PIF.

As part of a collaborative investigation of the association of medical students’ professional identity formation with their use of social media [22], the Consortium on Medical Education Research in Texas (Co-MERiT) sought measures of PIF to incorporate into a survey of students at nine Texas medical schools. Finding none appropriate for that use, the authors, in consultation with experts in identity fusion, undertook the development of a PIF assessment tool. Building on the work of Swann et al. [20], a pictorial representation of identity fusion was chosen. Previous work indicated, at least with respect to racial and ethnic identity, that the single pictorial measure captured the same variance and relationship with other variables as did a multi-item survey. Given students’ burden with respect to assessments and evaluations, a simple measure had considerable intuitive appeal. The purpose of this study was to assess the usefulness and validity of measures of identity fusion, using a simple pictorial-response format, as a facet of Professional Identity Formation (PIF). We hoped to illuminate how the measures functioned in some detail and how they might relate to the evolving concept of PIF.

### The measures

**Figure 1.**
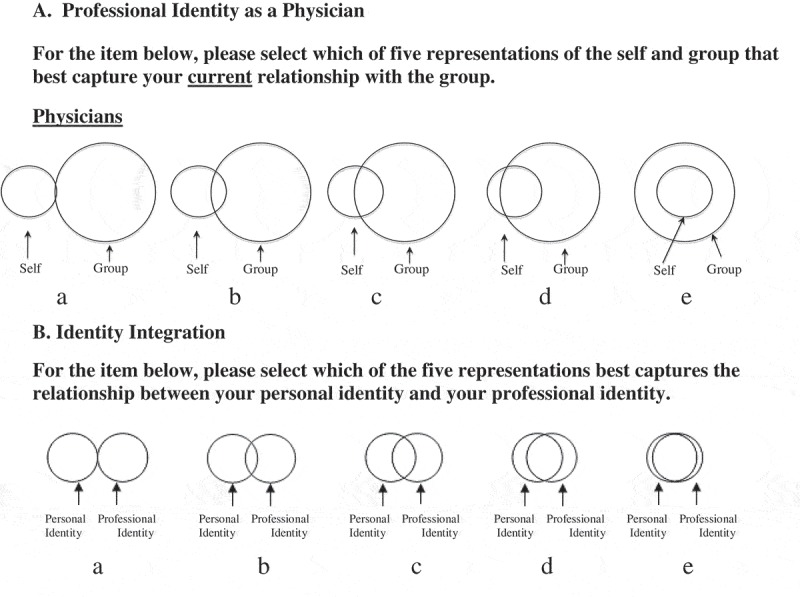
The Professional Identity as a Physician (PPI) measure (A) and the Identity Integration (IdIn) measure (B).

We examined responses to two pictorial measures. The first was part of a multi-item survey in the same format; the other was a single item presented in the context of a survey about students’ social media use. Both surveys were presented in a single administration. The multi-item survey included a measure of identity with physicians we named Physician Professional Identity (PPI) (see Figure 1). All items’ response options in the survey with the PPI item used a pictorial representation of varying amounts of overlap between self and an identified group. Ten items appeared as a set for groups relevant to possible identity fusion of medical students. These were: family, college alumni, medical school, medical school class, US medical students, physicians, race/ethnic group, religious group, personal online network, and professional online network. All items used the pictorial format seen in Figure 1 and were presented in the order listed. We chose those identity groups to allow examination of the pictorial-response format’s functioning (e.g., sufficiency of response variance). The item of interest asked about students’ identity with physicians (PPI).

The second measure, also a single item, asked about the fusion of personal and professional identities (Identity Integration – IdIn) (Figure 1). This measure used a pictorial-response format similar to that of the first measure. We developed this item as a result of students’ comments during preliminary pilot testing of survey items indicating they thought it would be a useful way of expressing the relationship of personal and professional identities. It was separated from the multi-item survey in which the PPI item appeared by 30 questions in various formats about students’ social media use. Thus, it appeared within a set of questions about the standards to which medical students should be held regarding social media use and how behavior differed in personal and professional contexts.

### Measure development and pilot testing

Following IRB approval at Texas A&M University Medical School, the multi-item survey draft, including the PPI item, was pilot-tested with 16 students (representing all four medical school years) and 11 internal medicine residents (representing interns, PGY2 and PGY3 residents). This group represented a breadth of experience and development representative of our target population and comprised individuals available within the development phase. The respondents first completed the survey. Then, researchers conducted structured interviews, asking respondents how they interpreted each item, particularly whom they thought about as a reference for each group, and how they interpreted the pictorial-response format. The time needed for survey completion was noted. Items for which there was low respondent consensus about interpretation were deleted to minimize survey fatigue. Survey instructions were rewritten for additional clarity. It was noted that the order in which items were presented might be important, as the first item (family) was cited by respondents as a standard to which subsequent items’ relationships were compared. Finally, as a result of respondents’ comments about integrating personal and professional identities, we developed the IdIn item as a separate measure embedded within a larger survey about social media use. Both measures were reviewed prior to by implementation for content appropriateness and usability by medical educators from Co-MERiT schools.

### Validity considerations

We used the modern construct of validity as a unitary concept [23,24] and therefore sought to gather evidence from multiple sources to support score interpretation. Because the construct of PIF is still being explicated, we generated for this work a set of broad hypotheses about construct validity and the impact of response format against which to examine evidence relative to the PPI and IdIn scores. Investigations were developed for each hypothesis to gather evidence for the trustworthiness of the scores as measures of medical students’ professional identity formation (PIF) (Table 1).

**Table 1.** Hypotheses for validity evidence.

*Hypotheses applying to both PPI and IdIn.*
H1: PPI and IdIn scores will have a strong positive association. Rationale: The constructs represented by PPI and IdIn scores were expected to be similar but not identical.
H2. PPI and IdIn scores will increase across years of training. Rationale: As identity formation is a developmental process^10^, we expected increases in scores for both measures with increases in training and/or maturation. (Increasing median scores would hold promise for being able to measure change in PIF with the scores.)
H3. Scores on a survey item addressing a related construct (indicate agreement with the statement: ‘What I do personally reflects on me as a professional’) will be positively related to PPI and IdIn scores. Rationale: Students who have a stronger identity with physicians and/or students with greater integration of personal/professional identities would have stronger agreement with this item; the associations should be stronger with increased level of training.
H4. Scores on a differently-formatted survey item addressing a related construct (yes/no to ‘A medical student should be held to higher standards than *other types* of students regarding the image he/she portrays on his/her personal [social media] page.’) would be positively related to PPI and IdIn scores. Rationale: If the pictorial response option format of the PPI and IdIn items did allow measurement of professional identity, higher scores on both PPI and IdIn would be associated with a ‘yes’ response (different response format) to this item.
*Hypothesis only for Physician Professional Identity measure (PPI).*
H5. There will be no relationship between PPI scores and responses to an unrelated but identically-formatted item about another identity construct. Rationale: It is reasonable to expect no statistical relationship between scores on identification with a religious group and PPI scores collected with the pictorial response format if that shared response format has no effect on the scores. A strong relationship between items directed at unrelated identity constructs, would suggest that the pictorial response format might be exerting response demand, calling into question the score validity.
*Hypothesis only for Identity Integration measure (IdIn).*
H6.There will be a negative relationship between IdIn scores and level of agreement with a related but logically-reversed survey item (‘My professional identity is different than my personal identity’). Rationale: If a negative association of moderate size is observed, concerns about the impact of response format on IdIn scores are reduced.

### Methods

The validation studies occurred within the context of a larger collaboration among nine Texas medical schools in the Co-MERiT partnership. The partnership study addressed primary research questions about medical students’ use of online social media (e.g., Facebook) and possible predictors or moderators of online behavior among medical students, including professional identity development. The social media study is reported elsewhere [22] and provided the context for data collection to address validity questions.

All schools agreed to the core items and data collection methods that were feasible at the individual schools (some online, others in person). Eight schools received IRB approval to collect and share data (Baylor College of Medicine, University of Texas Medical Branch, McGovern Medical School, University of Texas Southwestern, Texas A&M University, Texas Tech Paul L Foster School of Medicine, University of North Texas College of Medicine, University of Texas School of Medicine at San Antonio). Collaborating schools administered surveys in a variety of ways, including large group administrations, seeking volunteers at school events, and online solicitation.

All schools’ data were pooled, cleaned and analyzed in a central office. Response options for both PPI and IdIn items were converted from letters A to E to numerals 1 to 5, providing an ordinal scale whose higher numbers indicated a greater overlap of the circles in PPI and IdIn (Figure 1). Descriptive statistics were reviewed for anomalies and to determine if the responses could be considered representative of students from all nine schools. Analyses were conducted for each validity hypothesis (H1-H6) in Table 1. Spearman’s rho was chosen as the statistic for many of the analyses and is a non-parametric measure of association with a range of values −1 to +1. We considered values below ±.3 to be indicative of a weak association; ±.3 to ±.6, moderate; above ±.6, strong.

H1: To examine the association of PPI and IdIn scores, response frequencies and medians were used to characterize their distributions and variances. Spearman’s rho was calculated to estimate the association of the two score sets.

H2: To examine whether PPI and IdIn scores changed with level of training (maturation), we examined the median scores and response patterns by cohort (defined as year of training in medical school) for differences.

H3: Spearman’s rho was calculated to estimate the association of both PPI and IdIn scores with scores on an item the embedded in the larger survey, ‘What I do personally reflects on me as a professional.’ Scores for this item were derived from a 6-option response coded 0 − 5 (Strongly Disagree to Strongly Agree). The relationships were analyzed by cohort to examine strength of association as training level increased.

H4: Cramer’s V, a Chi square-based statistic, was calculated to estimate the association of PPI and IdIn scores with responses to a yes/no question about whether medical students should be held to higher standards than other students regarding personal social media sites (e.g., Facebook). The Cramer’s V statistic accommodates nominal variables and adjusts for different sample sizes in groups; it results in a common scale, 0 to 1, for association, allowing comparison of the degree of association for PPI and IdIn scores with the yes/no responses. Unlike measures of association for ordinal and continuous variables, however, it does not indicate the association’s direction.

H5: To investigate whether response patterns might be due to the pictorial-response format rather than true variation in constructs of interest, Spearman’s rho was calculated to test for association between PPI and religious identity scores, one of the group-identity items presented in the multi-item survey but believed to be unrelated to PIF. Mean scores for religious identity were examined for an increasing trend across cohorts.

H6: Spearman’s rho was used to measure association between an item with a six-option response scale (‘My professional identity is different than my person identity’) and IdIn scores. Both items addressed personal and professional identity fusion. Analyses of association were carried out for each cohort.

### Results

Data were provided by 8 of the 9 consortium schools, comprising responses from 3473 students. There was one osteopathic school among those providing responses. The data were unevenly distributed among schools and student cohorts (Table 2). Response rates varied by school, with between 17.3% and 94% of eligible students in each cohort participating. The schools’ mean response rate was 60.04% per cohort. Medical school years (MSY) 3 and 4 students (clinical years) were less well represented than MSY-1 and −2 students (preclinical years). Overall, 48.5% of respondents were female; 69.8% reported never being married; 64.4% were in the 20–25 age range. The number of students and response rates were deemed sufficient to proceed with planned analyses.

Hypothesis 1: Expectation of strong positive association between PPI and IdIn scores

**Table 2.** Response distribution for 3473 students at 8 schools.

MSY	SchoolA	SchoolB	SchoolC	SchoolD	SchoolE	SchoolF	SchoolG	SchoolH	Total N
1	101/206	214/228	178/212	80/178	182/230	48/150	178/234	167/257	1148/1695
	49%	94%	84%	45%	79%	32%	76%	65%	68%
2	109/195	145/158	178/225	112/175	156/229	33/150	173/240	152/234	1058/1606
	56%	92%	79%	64%	68%	22%	72%	65%	66%
3	69/160	48/133	NC	NC	170/230	26/153	160/235	196/236	669/1148
	43%	36%			74%	17%	68%	83%	58%
4	51/150	39/98	NC	116/176	NC	30/143	170/230	192/237	598/1033
	34%	40%		66%		21%	74%	81%	58%
Total	330/711	446/616	356/437	308/529	508/690	137/596	681/940	707/964	3473/5482
	46%	72%	81%	58%	74%	23%	72%	73%	63%

Legend: Topline in each cell contains: number of responses/total number surveyed; lower line is the cohort’s response rate. (NC = Not Collected)

The entire 1–5 response range of scores was represented in both PPI and IdIn scores. The median PPI score was 2.0 (interquartile range, IQR, = 1). The median IdIn score was 4.0 (IQR = 1). The Spearman’s rho statistic across all respondents was .11 and within cohorts were .10 (MSY-1), .15 (MSY-2), .12 (MSY-3) and .11 (MSY-4), with p < .05 for all. These weakly positive associations between PPI and IdIn scores did not support a claim that PPI and IdIn scores represented similar or overlapping constructs.

Hypothesis 2: Expectation of higher PPI and IdIn scores (median responses) as year of training increases

**Figure 2.**
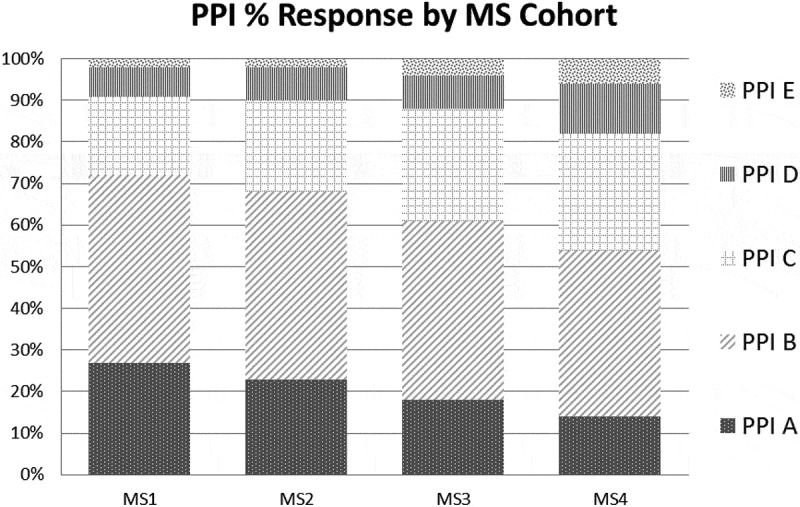
For the PPI item, the response pattern for the five options on the scale in Figure 1 varies across cohorts of medical students.

**Figure 3.**
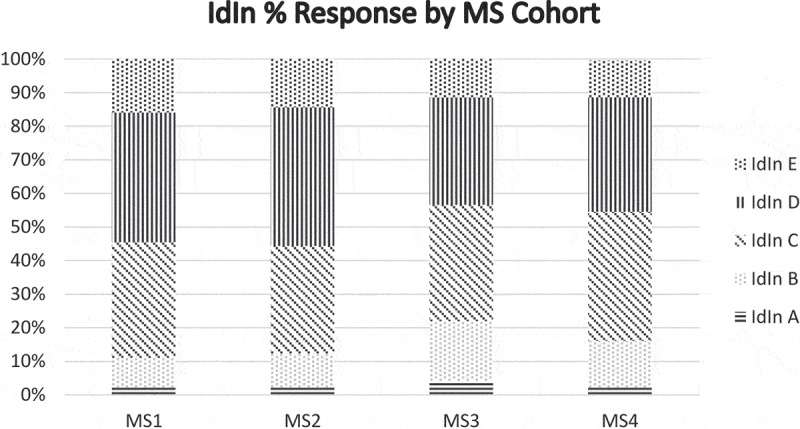
For the IdIn item, the response pattern for the five options on the scale in Figure 2 varies across cohorts of medical students.

The median PPI score across all cohorts was 2. The median IdIn score for MSY-1, MSY-2, and MSY-3 was 4, while the median IdIn score for MSY-4 was 3. The PPI score distribution (Figure 2) revealed slightly increasing proportions of students indicating higher levels of identity with physicians across cohorts, providing some evidence supporting score interpretation as representing a developmental process of identity formation. The slight shift in IdIn score distributions (Figure 3) was notable for the difference between preclinical and clinical years in the opposite direction of that expected.

Hypothesis 3: Expectation of positive relationship between PPI and IdIn scores and scores on first construct-relevant survey item

This set of analyses examined the association of agreement scores on the item ‘What I do personally reflects on me as a professional’ and the PPI and IdIn scores. The Spearman’s rho statistics (Table 3) indicated a very weak association between the item responses and PPI scores but a moderately positive relationship with IdIn scores. No trends were apparent across cohorts. The IdIn score analyses provided some moderately strong evidence supporting a claim that the IdIn item addressed an aspect of PIF. The PPI score analyses did not support a similar claim for that item.

Hypothesis 4: Expectation of positive relationship between PPI and IdIn scores with ‘yes’ responses to second construct-relevant survey item

**Table 3.** Association (Spearman’s rho statistics) of PPI and IdIn scores with responses to ‘What I do personally reflects on me as a professional’ (Hypothesis 3).

Measure	MS1	MS2	MS3	MS4
PPI	.01	.02	.03	.04
IdIn	.34	.31	.33	.34

All correlations p < .05.

Students responded yes or no (scored 1 or 0) to a question about whether medical students should be held to a higher standard than other students regarding their behavior on social media. Cramer’s V statistics were calculated to examine whether the yes/no responses were associated with scores for PPI and IdIn. The results (Table 4) indicated that PPI scores had no or very weak association with responses for this question; IdIn scores had a larger but still small positive association with the yes/no responses across all four cohorts of students. These analyses provide some weak support for interpreting IdIn scores in the context of PIF but none for PPI scores.

Hypothesis 5: Expectation of no relationship between PPI scores and scores on construct-irrelevant survey item with same response format

**Table 4.** Association (Cramer’s V statistics) of PPI and IdIn scores with yes/no response to the statement that medical students should be held to higher standards than other students for social-media behavior (Hypothesis 4).

Measure	MS1	MS2	MS3	MS4
PPI	.09	.11*	.15*	.13*
IdIn	.25**	.17**	.21**	.27**

*p < .05

**p < .0001

Responses across all respondents about identity with a religious group had a median of 3 (IQR = 3); the full range of possible responses was observed for this item. The item’s modal response was 1, with 30% of students choosing that option and the remainder spread evenly (16%-18%) across the remaining four response options. Cohort median scores for identity with a religious group were 3 for MSY-1, MSY-2, and MSY-3; for MSY-4, the median score was 2. This score distribution pattern and variance differed from those seen in PPI scores, particularly in the larger IQR. The Spearman’s rho coefficient between PPI scores and the identity with religious group scores across all cohorts was .16 (p < .02), a weak positive association. This investigation provides modest evidence that PPI scores are independent of the response format.

Hypothesis 6: Expectation of negative relationship between IdIn scores and scores on construct-relevant item presented in logically-reversed form

The Spearman’s rho coefficient for the association between IdIn scores and scores for level of agreement with the statement, ‘My professional identity is different than my personal identity’, calculated by cohort, were moderately negative: −.50 for MSY-1, −.47 for MSY-2 and MSY-3, and −.50 for MSY-4, with p < .05 for all correlations. These results providing reasonably strong evidence that IdIn scores represent integration or fusion of personal and professional identities.

### Discussion

Study respondents were representative of the medical student population in the state in age, gender, and marital status. Clinical year students were less well represented than preclinical students, but numbers adequate for the study’s purposes were obtained. We have learned both about the function of these measures and their association with

Our expectations that scores on the two measures of PIF being investigated, PPI and IdIn, would be moderately positively correlated were not met (Hypothesis 1). Because the whole-sample IdIn scores had a median of 4 and PPI scores of 2, the opportunity for variance in either item across cohorts was limited. It is possible that the pictorial-response scale was not sensitive enough to detect actual covariance in PPI and IdIn constructs. The two items may have different meanings such that they would logically elicit scores that do not covary: PPI addresses identity with physicians as a group, while IdIn was worded to address the integration of an individual’s personal and professional identity. First and second-year students’ ability to integrate what is likely a largely yet-unformed professional identity may also have been particularly difficult to measure. Both constructs may be important in the process of PIF, but in this study, the associated scores did not vary together in a linear fashion; this suggests the constructs represented by PPI and IdIn may not be the same. We recommend investigations of the relationship between these constructs as identities develop. Longitudinal studies will be necessary to characterize development across years of study.

Differences among cohorts revealed a modest trend in the expected direction for PPI scores, but not for IdIn scores (Hypothesis 2). The cohort differences for PPI scores were less than would be useful for comparisons such as those needed to evaluate curricular innovations’ effect on PIF or to identify students requiring additional support. The use of the PPI measure with other PIF measures may prove effective. The lack of cohort differences in IdIn scores is interpreted as a lack of evidence supporting this item’s use in assessing the developmental aspects of PIF during undergraduate medical education. One possible interpretation of differences in the distribution of IdIn scores would be that students may experience professional identity in a different way during the clinical years, finding the emergence of a new aspect of identity to be somewhat less integrated with an existing personal identity during this time. Studies of the impact of early clinical experience on PIF would be enlightening [25].

Agreement with the statement ‘What I do personally reflects on me as a professional’ was unrelated to the PPI scores and moderately related to IdIn scores (Hypothesis 3). This evidence supports the claim that the construct behind IdIn scores is integration of personal and professional identities and adds to the evidence that the PPI and IdIn measures may assess different constructs or different aspects of the overarching PIF construct.

Agreement with the statement that medical students should be held to higher standards for social-media behavior was positively related to scores on both PPI and IdIn, although more strongly to the IdIn scores. (Hypothesis 4) The pattern of agreement across cohorts provided supportive evidence that the PPI and IdIn scores were assessing PIF.

The performance of the item on identity with a religious group provided evidence that responses to pictorial-response items were free from influence attributable to that response format (Hypothesis 5). The difference in the scores’ variance provides evidence supporting the hypothesis, but the weakly positive estimate of the association between the two sets of scores is more difficult to interpret. It may be fruitful to conduct qualitative studies to investigate the role, if any, of religious identity in professional identity formation.

The expected negative relationship of IdIn scores and agreement with a statement that ‘My professional identity is different than my personal identity’ (Hypothesis 6) provides strong evidence for the IdIn construct. Respondents who replied ‘no’ to the latter item were more likely to have higher IdIn scores. This finding supports a claim that the pictorial-response format did not introduce construct-irrelevant variance, a common threat to validity.

In summary, the search for validity evidence for PPI and IdIn scores as measures of PIF in medical students resulted in mixed findings (Table 5), a not unexpected result given the nascent nature of work on the assessment of PIF. Continued study of these measures of PIF is clearly required. The accumulated evidence suggests that the scores do represent constructs within Professional Identity Formation. This represents a new application of the theoretical work in psychology regarding identity fusion [21]. The unified approach to validity seeks not to establish the presence or absence of validity but to consider how much validity evidence exists to support score interpretation. We found sufficient evidence for the validity claims to suggest that these measures assess aspects of PIF and may provide useful information as a component of an approach to the assessment of that larger construct.

**Table 5.** Summary of validity evidence for PPI and IdIn scores.

Hypothesis	Evidence for PPI scores	Evidence for IdIn scores	Summary for validity hypotheses
H1	None	None	Unconfirmed
H2	Modest	Unclear	Modest for PPI
H3	None	Moderate	Moderate for IdIn
H4	Modest	Moderate	Some evidence for both measures
H5	Moderate	– –	Confirmed
H6	– –	Strong	Confirmed

This study included only medical students, despite the likelihood that PIF processes extend well into physicians’ practice years. Continued study should include an examination of how these two measures perform longitudinally by extending investigations into graduate medical education and with practicing physicians. Until these studies are satisfactorily completed, we recommend caution when interpreting PPI and IdIn scores, using them as part of a multidimensional approach to the assessment of PIF while PIF itself is clarified and its assessment refined. PPI should be reported as a measure of identification with physicians and IdIn as identity integration, both of which may be aspects of Professional Identity Formation. Investigators should consider that PIF may occur in a non-linear fashion, requiring longitudinal designs that allow for non-linear analyses. We also recommend mixed-methods investigations that include these measures with qualitative approaches to move this research domain forward.

The study’s cross-sectional design limited our findings. While we were willing to assume that 4^th^-year students’ professional identities would be more developed than those of 1^st^-year students, the developmental rate and trajectory are still largely unknown. Longitudinal investigations are required to establish those characteristics of PIF. Further, our respondent group had fewer 3^rd^ – and 4^th^-year students than students in earlier training years, so our design was likely less sensitive than desired to changes occurring across years. It is also possible, however, that undergraduate medical education is not the phase during which significant developmental changes in PIF happen, e.g., perhaps students do not shed their ‘student’ identities until after completing medical school. Following individuals over time through undergraduate medical education and into residency and practice will be necessary to achieve deep understanding of PIF and to refine its measures. Investigations such as the ones reported in this paper are, however, useful initial steps.

The lack of well-developed assessment methods for professional identity, as well as the complexity of the constructs involved, present significant measurement challenges. Professional Identity Formation (PIF) is a complex and dynamic process, likely somewhat idiosyncratic with its course depending on an individual’s experience and style. The complexity of the process may, in the end, be found to resist simple measures, no matter how intuitively appealing those measures may be. The current lack of standards and deep understanding of PIF are obstacles to the development of elegant measurement solutions. The work described in this paper contributes to the understanding of PIF as a construct, however, and to conversation about its assessment. These measures track a single thread in a complex tapestry as existing personalities are transformed through an extensive socialization process to construct new personal and professional identities [6].

Future work in these areas must continue to consider the validity of potential measures’ scores for assessing PIF. We agree with Norcini and Shea [15], that we must move beyond self-report to assessing actual behavior in clinical settings. This paper offers the seed of an assessment strategy that may prove useful in combination with other approaches or in other contexts or with modification. The pictorial-response format is promising. We found some evidence supporting validity claims for scores from both measures investigated. Future investigators should carefully expand our understanding of the PIF construct while continuing to refine trustworthy measures.
